# A flexible electron-blocking interfacial shield for dendrite-free solid lithium metal batteries

**DOI:** 10.1038/s41467-020-20463-y

**Published:** 2021-01-08

**Authors:** Hanyu Huo, Jian Gao, Ning Zhao, Dongxing Zhang, Nathaniel Graham Holmes, Xiaona Li, Yipeng Sun, Jiamin Fu, Ruying Li, Xiangxin Guo, Xueliang Sun

**Affiliations:** 1 Department of Mechanical and Materials Engineering, University of Western Ontario, London, ON N6A 5B9 Canada; 2 State Key Laboratory of High Performance Ceramics and Superfine Microstructure, Shanghai Institute of Ceramics, Chinese Academy of Sciences, 200050 Shanghai, China; 3 State Key Laboratory of Organic-Inorganic Composites, Beijing University of Chemical Technology, 100029 Beijing, China; 4 College of Physics, Qingdao University, 266071 Qingdao, China; 5 Department of Chemistry, University of Western Ontario, London, ON N6A 5B9 Canada

**Keywords:** Batteries, Batteries, Batteries

## Abstract

Solid-state batteries (SSBs) are considered to be the next-generation lithium-ion battery technology due to their enhanced energy density and safety. However, the high electronic conductivity of solid-state electrolytes (SSEs) leads to Li dendrite nucleation and proliferation. Uneven electric-field distribution resulting from poor interfacial contact can further promote dendritic deposition and lead to rapid short circuiting of SSBs. Herein, we propose a flexible electron-blocking interfacial shield (EBS) to protect garnet electrolytes from the electronic degradation. The EBS formed by an in-situ substitution reaction can not only increase lithiophilicity but also stabilize the Li volume change, maintaining the integrity of the interface during repeated cycling. Density functional theory calculations show a high electron-tunneling energy barrier from Li metal to the EBS, indicating an excellent capacity for electron-blocking. EBS protected cells exhibit an improved critical current density of 1.2 mA cm^−2^ and stable cycling for over 400 h at 1 mA cm^−2^ (1 mAh cm^−2^) at room temperature. These results demonstrate an effective strategy for the suppression of Li dendrites and present fresh insight into the rational design of the SSE and Li metal interface.

## Abstract (web summary)

The high electronic conductivity of solid-state electrolytes leads to Li dendrite growth, thus hindering the commercialization of solid-state batteries. Here, the authors propose a flexible electron-blocking interface to protect garnet electrolytes from the electronic degradation.

## Introduction

Due to the rapid development of portable devices and electric vehicles, current lithium-ion batteries cannot meet future requirements for energy density, cycle life, and safety^1^. Solid-state batteries (SSBs) have received much attention for their potential as next-generation batteries^2^. Solid-state electrolytes (SSEs) paired with a Li metal anode and a high-voltage cathode not only enhance energy density but also improve safety through the elimination of flammable liquid electrolytes.

Many SSEs have been reported, including lithium phosphorus oxynitride (LiPON)^3^, sulfide-type^4^, sodium superionic conductor (NASICON)-type^5,6^, perovskite-type^7^, halide-type^8^, and garnet-type^9^. Among them, garnet-type Li_7_La_3_Zr_2_O_12_ (LLZO) is promising because of its excellent chemical/electrochemical stability with Li metal and its high ionic conductivity at room temperature^10,11^. LLZO has a high shear modulus (~55 GPa) and Li transference number (~1), and was therefore predicted to prevent Li dendrite growth based on Sand’s theory^12–15^. Unfortunately, numerous studies have reported garnet electrolytes suffering from serious Li dendrite penetration through grain boundaries^16^, pores^17^, and even through single crystals^18^.

It is generally acknowledged that interfacial properties play a critical role in regulating Li deposition^19^. LLZO shows poor wettability with Li metal. Large interfacial resistance which promotes Li dendrite nucleation is therefore difficult to prevent. Various strategies have been proposed to enhance the interfacial contact between LLZO and Li metal, such as introducing intermediate layers^20–22^, cleaning surface contaminants^23,24^, increasing the pressure or temperature^25,26^, and constructing a three-dimensional (3D) interfacial structure^27^. These approaches improve the wettability and thus reduce Li dendrite propagation to some extent; however, lithium penetration through the electrolyte still occurs with increased current density or extended cycling time^28^. These results indicate that improvement of interfacial contact is not enough to address dendritic deposition on its own.

Although the mechanisms for Li dendrite growth remain elusive, the electron attack to the garnet electrolytes has recently received increased scrutiny since high electronic conductivity of SSEs was reported as the cause for certain types of Li dendrites^29^. The electron attack to the garnet electrolytes was recently visualized by a scanning electron microscope (SEM)^30^. The electron beam in the SEM irradiated on the Ta-doped LLZO (LLZTO) surface can expulse the Li out of the LLZTO to satisfy the neutrality. Due to heterogeneous Li^+^ transport in LLZTO electrolytes, Li^+^ preferentially accumulates in defects and voids, forming metallic Li as it combines with electrons^31,32^. Poor interfacial contact leads to an uneven electric field, resulting in large local currents at the interface and promoting rapid Li dendrite penetration through the electrolyte. The construction of an electron-blocking interface with excellent wettability is therefore important for the development of dendrite-free Li anodes in SSBs^33^. Unfortunately, most interlayers alloy with Li metal and are electronically conductive, while ionically conductive but electronically insulating materials show large interfacial resistance with Li metal^34^. A hybrid interlayer formed with electronically conductive nanoparticles embedded in an ionically conductive matrix was recently reported to achieve excellent interfacial wettability and a uniform electric field distribution^28^; however, the improvement of electrochemical performance was still limited due to the conductive interface not preventing electron mobility within the garnet electrolyte.

Herein, we propose a flexible electron-blocking interfacial shield (EBS) to achieve uniform interfacial contact and prevent dendritic deposits attributed to high electronic conductivity in garnet electrolytes. Polyacrylic acid (PAA) polymer at the interface reacts with molten Li at 250 °C, forming Li-inserted PAA (LiPAA). Such an EBS leads to good wettability with Li metal, decreasing the interfacial resistance from 1104.3 to 54.5 Ω cm^2^ at 25 °C. In addition, the flexible polymer interface relieves the interfacial stress generated by the changing volume of the Li anode, thus maintaining excellent interfacial contact during cycling^35^. The electron-blocking nature of the EBS is supported by density functional theory (DFT) calculations. Electrostatic potential profiles and density of states (DOS) profiles show that the LLZTO electrolyte and surface Li_2_CO_3_ contamination conduct electrons, while the EBS is electrically insulating. As a proof of concept, garnet electrolytes with EBS show improved performance in both Li symmetric cells and LiFePO_4_/Li cells.

## Results

### Characterizations of the LLZTO@PAA

LLZTO ceramic pellets were fabricated by the hot-press sintering technique detailed in our previous study^36^. Cross-sectional SEM images of LLZTO show a transgranular fracture morphology without obvious grain boundaries, leading to a high relative density of over 99.5% (Supplementary Fig. 1a)^37^. The X-ray diffraction (XRD) pattern, shown in Supplementary Fig. 2, shows diffraction peaks which match well with the standard pattern of cubic-phase garnet electrolytes (PDF#45-0109). The high relative density and pure cubic phase result in an ionic conductivity as high as 1.1 × 10^−3^ S cm^−1^ at 25 °C (Supplementary Fig. 1b). PAA exhibits an amorphous structure with a broad peak at 2*θ* = ~18°^38^. The PAA was dissolved in a dimethyl sulfoxide (DMSO) solution and coated on the surface of the LLZTO by drip casting. To evaluate the chemical stability between the LLZTO, PAA, and DMSO, the LLZTO particles were mixed with the PAA slurry and the DMSO solvent evaporated at 80 °C. The XRD pattern in Supplementary Fig. 2 shows no change to the garnet structure, confirming the stability of the constituent components.

**Fig. 1:**
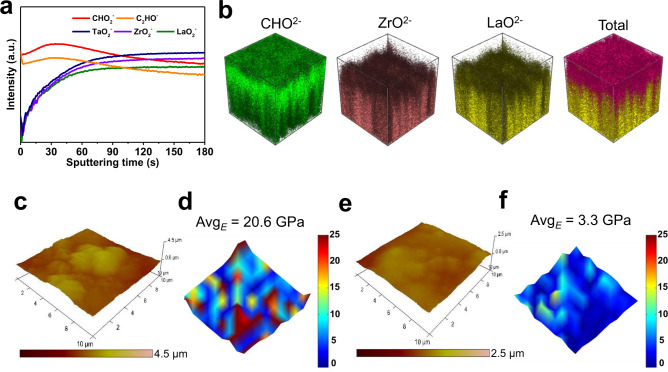
Physical properties of the PAA layer on the surface of LLZTO. **a** TOF-SIMS depth profiles for the LLZTO@PAA pellet. **b** 3D views of the sputtered volume of LLZTO@PAA. **c** AFM topography image, and **d** Young’s modulus of LLZTO. **e** AFM topography image, and **f** Young’s modulus of LLZTO@PAA.

Time-of-flight secondary-ion mass spectroscopy (TOF-SIMS) was carried out to examine the thickness and homogeneity of the PAA thin films on garnet electrolytes. TOF-SIMS depth profiling reveals the composition of fragments from the specimen during the sputtering process^39^. Here, CHO_2_^−^ and C_2_HO^−^ fragments originate from the PAA layer, while LaO_2_^−^, ZrO_2_^−^, and TaO_2_^−^ fragments come from the LLZTO underneath. As shown in Fig. 1a, the CHO_2_^−^ and C_2_HO^−^ signal intensities are initially high, but gradually decline after 45 s of Cs^+^ sputtering. In contrast, the LaO_2_^−^, ZrO_2_^−^, and TaO_2_^−^ signals from the LLZTO initially weak, but gradually increase during the 45 s of Cs^+^ sputtering. A uniform PAA film is thus shown to coat the LLZTO pellet. The thickness of the PAA coating is estimated to be 43 nm based on a sputtering rate of 0.96 nm s^−1^. Supplementary Fig. 3 shows the TOF-SIMS mappings of the CHO_2_^−^, C_2_HO^−^, LaO_2_^−^, ZrO_2_^−^, and TaO_2_^−^ signals after sputtering. Strong LaO_2_^−^, ZrO_2_^−^, and TaO_2_^−^ signals corresponding to LLZTO are observed from the sputtered region and intense CHO_2_^−^ and C_2_HO^−^ signals corresponding to the PAA are observed across the pristine region. Three-dimensional views of the sputtered volume of LLZTO@PAA directly visualize the homogeneous coverage of PAA on the surface of the LLZTO electrolyte (Fig. 1b). In addition, Supplementary Fig. 4 shows the cross-sectional SEM image and energy dispersion spectrum (EDS) scanning of the PAA-coated LLZTO pellet. The thickness of the uniform PAA film is ~48 nm, which is consistent with the result of TOF-SIMS.

Topographical atomic force microscopy (AFM) images indicate that loose surface contaminants (e.g. Li_2_CO_3_) from exposure to air leave LLZTO pellets with a rough surface (Fig. 1c)^40^. The surface becomes relatively smooth after coating with the PAA film (Fig. 1e). Interfacial hardness can greatly affect Li dendrite growth due to residual stresses during repeated cycling^41^. Interfaces with poor ductility may be broken by the Li volume change, leading to poor interfacial contact and large resistance^35^. A soft interface is therefore required to relieve interfacial stress and maintain good interfacial contact. To compare the surface hardness before and after coverage with PAA, Young’s modulus (*E*) mappings were created by fitting force–distance curves at 100 locations in a 30 × 30 μm^2^ area. The average Young’s modulus (Avg_*E*_) for LLZTO is 20.6 GPa, while the Avg_*E*_ for LLZTO@PAA is 3.3 GPa (Fig. 1d and f). The decreased Avg_*E*_ indicates a flexible interface which can serve as a stable interface during cycling and suppress Li dendrite growth.

### Formation of the EBS by the substitution reaction

The EBS was formed in situ by the reaction of a PAA film with molten Li at 250 °C. The reaction mechanisms and products were investigated using first-principles calculations. Supplementary Fig. 5a and b shows there are two possible reaction mechanisms between PAA and molten Li. One is a recombination reaction, where Li inserts directly into PAA polymer chains. The other is a substitution reaction, where Li replaces the H in a PAA -COOH group. The electrostatic potential profiles in Supplementary Fig. 5c show that the dehydrogenated interphase created by the substitution reaction is more stable. Differential electrochemical mass spectrometry (DEMS) was used to detect the H_2_ release and further confirm the substitution reaction (Supplementary Fig. 6 and Supplementary Note 1). The escaping electrons accompanied by H_2_ gas release suppress the interfacial electrostatic potential and prohibit electron permeation. The structure and composition of LiPAA were studied by SEM, X-ray photoelectron spectroscopy (XPS), Fourier Transform Infrared Spectroscopy (FTIR), and Raman, which can support the results of theoretical calculations (Supplementary Fig. 7 and Supplementary Note 2).

**Fig. 2:**
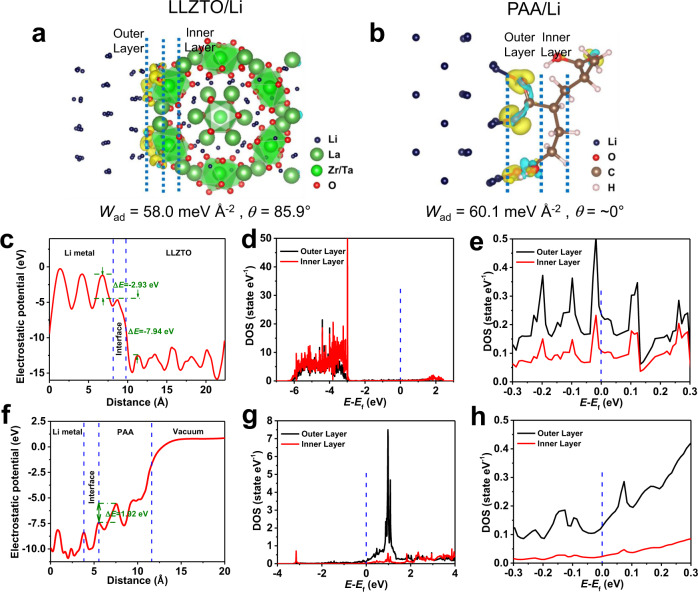
Electron-blocking property of the PAA-modified LLZTO with Li metal. **a** The structure and charge transfer, **c** the electrostatic potential profiles, and **d**, **e** the density of states (DOS) for fully relaxed LLZTO(110)/Li(001). **b** The structure and charge transfer, **f** the electrostatic potential profiles, and **g**, **h** the density of states (DOS) for fully relaxed PAA/Li(001) single chain (the vacuum slab is not entirely presented in the model).

The work of adhesion (*W*_ad_) for dehydrogenated PAA on Li metal is 60.1 meV Å^−2^, much higher than the 58.0 meV Å^−2^ for LLZTO(110)/Li(001) and the 16.5 meV Å^−2^ for Li_2_CO_3_(001)/Li(001) (Fig. 2a, b and Supplementary Fig. 8a). Note that Li_2_CO_3_ is the main component of the contamination on LLZTO surfaces exposed to air^40^. As a result, the PAA layer improves the wettability of Li metal on LLZTO, especially when the LLZTO is covered by lithiophobic Li_2_CO_3_. The contact angle was calculated with the following equation:

1 $$W_{{\mathrm{ad}}} = \sigma _{{\mathrm{Li}}}(1 + {\mathrm{cos}}\, \theta ) = \frac{{E_{{\mathop{\rm{int}}} {\mathrm{erface}}} - E_{{\mathrm{Li}} - {\mathrm{slab}}} - E_{{\mathrm{PAA(LLZTO/Li}}_{\mathrm{2}}{\mathrm{CO}}_{\mathrm{3}}{\mathrm{)}} - {\mathrm{slab}}}}}{S},$$

where *W*_ad_ is the interfacial work of adhesion, *σ*_Li_ is the surface energy of Li, and *θ* is the contact angle^42,43^. The *θ* for PAA/Li, LLZTO/Li, and Li_2_CO_3_/Li is ~0°, 85.9°, and 132.7°, respectively, indicating greatly improved wettability between the LLZTO and Li metal using a PAA intermediate layer.

### Electron-blocking property of the EBS

A LiPAA EBS effectively blocks electrons at the interface. This fact is confirmed by the electrostatic potential profiles and DOS simulation results shown in Fig. 2c and Supplementary Fig. 8b for LLZTO(110)/Li(001) and Li_2_CO_3_(001)/Li(001), respectively. There is no barrier to the transfer of electrons from the interface to the LLZTO electrolyte. In the case of LLZTO(110)/Li(001), electrons and Li atoms preferentially deposit within the LLZTO rather than at the LLZTO/Li interface, a behavior corroborated by DOS results. The result is that LLZTO becomes electronically conductive when lithiated (Fig. 2d and e), forming Li dendrites across LLZTO electrolytes^29^. The interfacial electron density of Li_2_CO_3_(001)/Li(001) is higher than that of LLZTO(110)/Li(001). An abnormal space charge layer is shown in Supplementary Fig. 8c and d. The outer layer has a slightly higher electronic DOS than the inner layer, indicating that insulative Li_2_CO_3_ promotes electron permeation due to complex interfacial phenomena. In contrast, the electrostatic potential of the PAA/Li(001) interface is 1.92 eV lower than LiPAA polymer, which is attributed to the dehydrogenation reaction (Fig. 2f). Electrons are contained to the Li metal and permeate only into the outer layer of the interface. In addition, Li deposition occurs preferentially at the interface rather than within the LiPAA, prohibiting the penetration of Li dendrites through the PAA. The electronically insulating nature is further confirmed by DOS results for PAA/Li(001), shown in Fig. 2g and h. Electrons are captured within the Li/PAA interfacial bonds, while the inner layer remains insulating. To further confirm the electronically insulating property of LiPAA, the electronic conductivity of the LLZTO and the LLZTO@EBS was evaluated by DC polarization at 0.1 V. As shown in Supplementary Fig. 9, the electronic conductivity of the LLZTO@EBS is smaller than that of the LLZTO, indicating the excellent capability of electron block by the EBS.

**Fig. 3:**
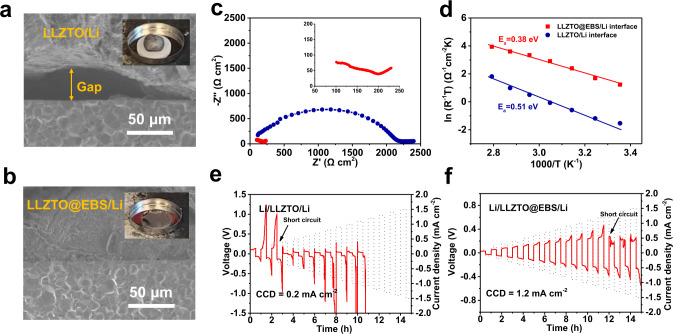
Comparation of interfacial wetting behaviors between bare LLZTO and PAA-modified LLZTO with molten Li. SEM images of **a** the LLZTO/Li interface and **b** the LLZTO@EBS/Li interface. Insets are the corresponding digital images showing the wetting behaviors of molten Li on bare LLZTO or LLZTO@EBS. **c** EIS spectra, **d** temperature-dependent interfacial resistance of the Li/LLZTO/Li and the Li/LLZTO@EBS/Li cells. **e** CCD of the Li/LLZTO/Li cell. **f** CCD of the Li/LLZTO@EBS/Li cell.

The LLZTO@EBS/Li wettability was evaluated with molten Li and LLZTO@PAA. As shown in Fig. 3a, molten Li forms a sphere on the LLZTO surface, indicating a large *θ*. This poor wettability leads to gaps at the interface. In contrast, molten Li completely wets LLZTO@EBS (Fig. 3b). Cross-sectional SEM image shows intimate contact between the LLZTO@EBS and the Li metal without any voids at the interface. This enhanced wettability is consistent with the simulated *θ*. Complete wetting significantly decreases the interfacial resistance, thus improving electrochemical performance.

### SSBs benefitting from the EBS

Li/LLZTO@EBS/Li and Li/LLZTO/Li symmetric cells were assembled for electrochemical characterization. Electrochemical impedance spectroscopy (EIS) was carried out to compare the interfacial resistance of cells with and without the EBS. Figure 3c shows the impedance spectra obtained at 25 °C. The impedance spectrum of the Li/LLZTO/Li cell exhibits one large semicircle. The starting point of the spectrum corresponds to the bulk resistance of the LLZTO, while the semicircle corresponds to the interfacial resistance between LLZTO and Li metal^37^. In an ideal situation, the charge transfer across two Li/LLZTO interfaces should be identical in a symmetric cell. The interfacial resistance determined from the semicircle is divided by two to obtain the value for each Li/LLZTO interface. Thus, the LLZTO/Li interfacial resistance is found to be 1104.3 Ω cm^2^. The Li/LLZTO@EBS/Li symmetric cell shows multiple semicircles resulting from the EBS bulk and EBS/LLZTO interface at high frequency and EBS/Li interface at low frequency. The overall resistance of the LLZTO@EBS/Li interface was 54.5 Ω cm^2^. The decrease in interfacial resistance from 1104.3 Ω cm^2^ to 54.5 Ω cm^2^ can be ascribed to the lithiophilicity of the EBS film. In addition, the temperature-dependence of the interfacial resistance was characterized between 25 °C and 85 °C. The activation energy (*E*_a_) of the EBS modified and the unmodified interface was calculated using the Arrhenius law. The *E*_a_ of the LLZTO@EBS/Li interface is 0.38 eV, while the *E*_a_ of the LLZTO /Li interface is 0.51 eV (Fig. 3d). The decreased *E*_a_ is beneficial for Li^+^ migration across the interface^28^.

The critical current density (CCD) was used as a measure of the interfacial stability and capacity for Li dendrite suppression. The CCD is defined as the current density where the cell reaches a short circuit. An applied current density was increased from 0.1 to 1.5 mA cm^−2^ with a step increase of 0.1 mA cm^−2^ per hour at 25 °C. Figure 3e shows that the CCD of the Li/LLZTO/Li cell is as low as 0.2 mA cm^−2^. The large overpotential over 1 V is a result of poor interfacial contact. The CCD of the Li/LLZTO@EBS/Li cell is significantly improved to 1.2 mA cm^−2^. The voltage profile of the Li/LLZTO@EBS/Li cell remains relatively stable before short circuiting. The improvement in CCD can be attributed to combined contributions from the electronically insulating interface and from the relieved interfacial stress. More specifically, the LiPAA EBS facilitates Li^+^ transport and prevents electronic degradation of the LLZTO bulk. In addition, the flexibility of the polymer interface alleviates interfacial stress, maintaining interfacial contact and suppressing Li dendrite growth. To our knowledge, a CCD of 1.2 mA cm^−2^ at room temperature is the highest value ever reported for garnet electrolytes (Supplementary Table 1). Despite the various surface modification approaches used to decrease interfacial resistance by enhancing wettability, the CCD is still limited due to electronic degradation and poor interfacial stability at high current densities.

**Fig. 4:**
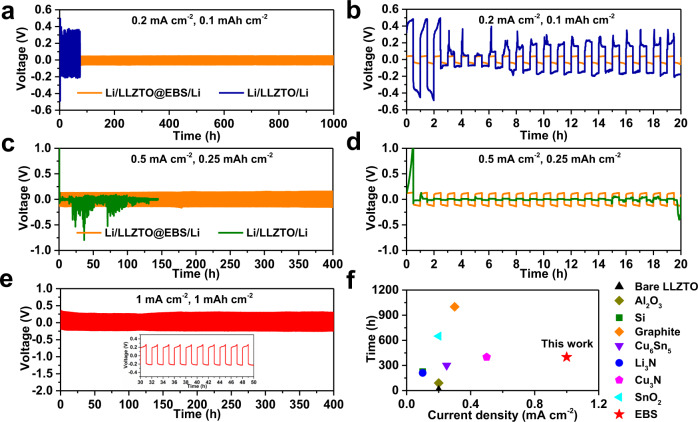
Electrochemical performance of Li symmetric cells using bare LLZTO and the PAA-modified LLZTO. **a** Galvanostatic cycling performance of the Li/LLZTO/Li and Li/LLZTO@EBS/Li cells at 0.2 mA cm^−2^ (0.1 mAh cm^−2^) at 25 °C; **b** Magnified images for 0–20 h. **c** Galvanostatic cycling performance of the Li/LLZTO/Li and Li/LLZTO@EBS/Li cells at 0.5 mA cm^−2^ (0.25 mAh cm^−2^) at 25 °C; **d** Magnified images for 0–20 h. **e** Galvanostatic cycling performance of the Li/LLZTO@EBS/Li cell at 1 mA cm^−2^ (1 mAh cm^−2^) at 25 °C. **f** Galvanostatic cycling performance in recent literature compared with our work^20,21,28,35,37,58,59^.

Galvanostatic Li plating/stripping experiments were carried out to evaluate the long-term stability of Li^+^ transport and the effectiveness of dendrite suppression at the interface. As shown in Fig. 4a, the Li/LLZTO/Li cell exhibits an overpotential over 0.45 V for the first charge/discharge cycle at 0.2 mA cm^−2^ (0.1 mAh cm^−2^), indicating inhomogeneous Li deposition. A short circuit occurs within three cycles. The poor LLZTO/Li interfacial contact leads to uneven current distribution and serious electronic degradation at the defects, thus inducing Li dendrite growth^44^. In contrast, the Li/LLZTO@EBS/Li cell continuously operates for over 1000 h with an overpotential of 46.1 mV at 0.2 mA cm^−2^ (Fig. 4b). Moreover, the Li/LLZTO@EBS/Li cell shows stable cycling for 400 h at 0.5 mA cm^−2^ (0.25 mAh cm^−2^), while the Li/LLZTO/Li cell cannot be cycled even once (Fig. 4c and d). Increasing the current density and areal capacity to 1 mA cm^−2^ and 1 mAh cm^−2^, the Li/LLZTO-EBS/Li cell continues to show stable cycling for 400 h (Fig. 4e). To our knowledge, the performance of LLZTO@EBS is superior to the performance achieved with garnet electrolytes in all previous studies (Fig. 4f).

**Fig. 5:**
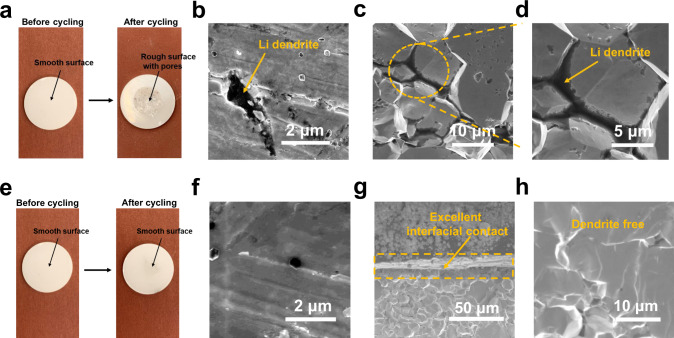
Li dendrite growth in the bare LLZTO and the PAA-modified LLZTO. **a** Optical surface morphologies of an LLZTO pellet before and after cycling. **b** Top-view and **c**, **d** cross-sectional SEM images of an LLZTO pellet after short circuiting. **e** Optical surface morphologies of an LLZTO@EBS pellet before and after cycling. **f** Top-view SEM image of an LLZTO@EBS pellet after cycling at 0.2 mA cm^−2^ for 1000 h. **g** Cross-sectional SEM image of the LLZTO@EBS/Li interface, and **h** cross-sectional SEM image of an LLZTO@EBS pellet after cycling at 0.2 mA cm^−2^ for 1000 h.

After disassembling the short-circuited Li/LLZTO/Li cell and reacting the Li metal with a water/alcohol solution, a rough LLZTO surface with voids and defects is revealed. The dark spots reveal areas where Li dendrites have grown into the LLZTO pellet (Fig. 5a). This is confirmed by SEM (Fig. 5b). The cross-sectional SEM image shows the proliferation of Li dendrites through the LLZTO grain boundaries (Fig. 5c and d), the cause of short circuiting. As shown in Fig. 5e and f, the surface of the LiPAA-protected LLZTO remains smooth after 1000 h without dark spots from dendrites. The flexible polymer EBS accommodates the Li volume change to maintain good contact (Fig. 5g). The slight increase in overall resistance from 209.1 to 224.3 Ω cm^2^ confirms that no short circuiting occurs after 1000 h of cycling (Supplementary Fig. 10). The dendrite-free grain boundary of the LLZTO further confirms the ability of the EBS to prevent dendrite growth (Fig. 5h).

## Discussion

**Fig. 6:**
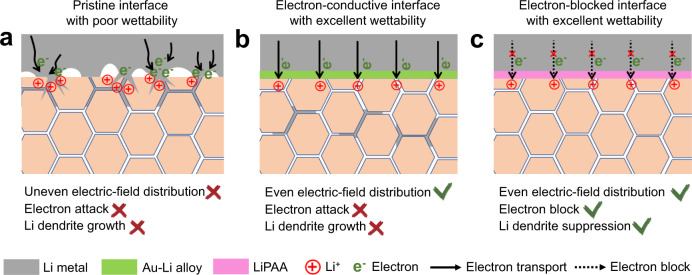
Schematic illustrations of Li dendrite growth at various interfaces. Schematic illustrations of Li dendrite growth at the **a** pristine LLZTO/Li interface, **b** LLZTO@Au/Li interface, and **c** LLZTO@EBS/Li interface.

To understand the mechanisms for dendrite suppression by the EBS, other interfacial layers were synthesized between the LLZTO electrolyte and Li metal for comparison. A Au layer was first coated on the LLZTO surface by sputtering. Supplementary Fig. 11a shows the excellent wettability of the LLZTO@Au with molten Li due to the formation of a Au–Li alloy at 300 °C. This enhanced wettability leads to a dramatically decreased interfacial resistance of 43.2 Ω cm^2^ (Supplementary Fig. 11b). The Li/LLZTO@Au/Li cell exhibits a CCD of 0.7 mA cm^−2^ and stable cycling over 200 h at 0.5 mA cm^−2^ (0.25 mAh cm^−2^). This is a marked improvement over the pristine Li/LLZTO/Li cell (Supplementary Figs. 11c and 12) where poor wettability of the LLZTO causes an uneven electric field and local hot spots which lead to Li dendrite nucleation and propagation (Fig. 6a). The improved cycling performance achieved with a Au interfacial provides a basis for the idea that interfacial wettability is important for the uniform distribution of the electric field (Fig. 6b).

Although the interfacial resistance of LLZTO@Au/Li is smaller than that of LLZTO@EBS/Li, the Li/LLZTO@Au/Li cell shows a much shorter cycle life than Li/LLZTO@EBS/Li cell at 0.5 mA cm^−2^ (Supplementary Fig. 12). This can be attributed to the electron attack^29^. Electrons within the electrolyte can combine with lithium ions to form Li metal within the polycrystalline electrolytes, especially at the grain boundaries. Pristine LLZTO with its lithiophobic nature accumulates electrons unevenly at locations with point contact, more readily forming Li dendrites (Fig. 6a). The LLZTO@Au is also unable to avoid this form of degradation due to the conductive nature of the interface (Fig. 6b). In contrast, the EBS protected LLZTO prevents electrons from entering the electrolyte, avoiding dendrite formation (Fig. 6c)^45^. The flexible LiPAA polymer maintains interfacial contact by accommodating the Li volume change during cycling. In contrast, the overpotential of the Li/LLZTO@Au/Li cell gradually increases after 150 h cycling due to fracturing of the interface, accelerating the short circuit (Supplementary Fig. 12).

To further explore the capability of the EBS layer in suppressing dendrites, PAA was coated on LLZTO electrolytes with a relative density of 96% (LLZTO(96%)). LLZTO(96%) facilitates Li dendrite growth compared to high-density LLZTO. The cross-sectional SEM image, shown in Supplementary Fig. 13a, shows that the LLZTO(96%) consists of small garnet grains with many grain boundaries and voids, leading to a decreased ionic conductivity of 5.4 × 10^−4^ S cm^−1^ at 25 °C (Supplementary Fig. 13b). The increased number of grain boundaries trap electrons and lead to increased Li dendrite formation^32,46^. Supplementary Fig. 14 shows short-circuiting of the Li/LLZTO(96%)@Au/Li cell after 18 h of cycling at 0.2 mA cm^−2^ (0.1 mA cm^−2^). SEM images show a large amount of mossy Li dendrite within the grain boundaries in the short-circuited LLZTO(96%) electrolyte (Supplementary Fig. 15). In contrast, the Li/LLZTO(96%)@EBS/Li cell demonstrates stable cycling for 150 h at 0.2 mA cm^−2^ (Supplementary Fig. 14).

Li^+^ transport at the interface also affects Li dendrite growth. For comparison, a traditional poly(ethylene oxide)/Lithium bis(trifluoromethanesulfonyl)imide electrolyte layer (PEO) was coated on the surface of LLZTO pellets. Supplementary Fig. 16a shows a PEO layer thickness of ~5 μm. The EIS spectrum for the Li/LLZTO@PEO/Li cell shows two semicircles at 60 °C (Supplementary Fig. 16b). The small semicircle at a high frequency corresponds to the resistance of the PEO layer, while the large semicircle at a low frequency is attributed to the resistance of the LLZTO/PEO and PEO/Li interfaces. The interfacial resistance resulting from the PEO modification is 683.2 Ω cm^2^ at 60 °C, one order of magnitude larger than the interfacial resistance of the EBS modification. Although the PEO layer is also electronically insulating, the sluggish Li^+^ transport at the interface leads to large overpotentials during the CCD test. The CCD of the Li/LLZTO/PEO/Li cell is 0.6 mA cm^−2^ (Supplementary Fig. 16c). In addition, polymer electrolytes with a Li salt as an intermediate layer always exhibit a low Li^+^ transference number (<0.5), which can induce uneven Li deposition^47,48^. In contrast, LiPAA polymer guides homogenous Li deposition without the interference of anions. The Li/LLZTO@PEO/Li cell shows an overpotential over 0.8 V and short circuits after 50 h of cycling at 0.5 mA cm^−2^ (0.25 mAh cm^−2^) at 60 °C (Supplementary Fig. 17). The Li/LLZTO@EBS/Li cell operates continuously for 400 h at 0.5 mA cm^−2^ (0.25 mAh cm^−2^) at 25 °C, indicating that the good Li^+^ transport at the EBS interface is beneficial to performance.

To extend the substitution reaction to other polymers, PEO film was coated on the surface of the LLZTO pellets and reacted with the molten Li by the same method. The interfacial resistance of the Li/LLZTO(PEO)/Li cell is even one order magnitude larger than that of the Li/LLZTO/Li cell without modification (Supplementary Fig. 18). The blocked Li^+^ transport could be attributed to the following reasons: (1) compared with the -OH in the PEO, -COOH of the PAA as an acid group is easier to react with the Li by the substitution reaction; (2) the -COOH group of the PAA is on the main polymer chain, while the -OH group of the PEO is the terminal group. This leads to a larger number of -COOH of the PAA for the substitution reaction and the Li^+^ transportation by the segment movement; (3) PAA (*M*_w_: ~450,000) shows the much smaller molecular weight than PEO (*M*_w_: ~1,000,000), which may be beneficial for the Li^+^ transport due to the decreased crystallinity.

Full SSBs with a LiFePO_4_ (LFP) cathode and a Li metal anode were constructed using LLZTO@EBS and compared to bare LLZTO. Supplementary Fig. 19a shows the configuration of the SSBs. The introduction of an ionic liquid as a wetting agent enhances Li^+^ migration into the composite cathode for room-temperature feasibility^23,40^. The LFP/LLZTO@EBS/Li cell shows smaller polarization than the LFP/LLZTO/Li cell at various current rates (Supplementary Fig. 19b and c). The LFP/LLZTO@EBS/Li cell delivers a specific discharge capacity of 142.3 mAh g^−1^ at 0.1 C. The discharge capacity is 130.2, 119.5, and 95.4 mAh g^−1^ at 0.2, 0.5, and 1 C, respectively (Supplementary Fig. 19d). After high-rate cycling, the cell can recover a discharge capacity of 142.5 mAh g^−1^ at 0.1 C. The high capacity and excellent cycling stability can be ascribed to good interfacial contact, an electronically insulating interface, and accommodation of the Li volume change. In contrast, the LFP/LLZTO/Li cell delivers a discharge capacity of 125.4 mAh g^−1^ with a high overpotential at 0.1 C. The discharge capacity decreases to 105.2, 79.6, and 49.2 mAh g^−1^ at 0.2, 0.5, and 1 C, respectively (Supplementary Fig. 19d). Moreover, the SSB with LLZTO@EBS retains 82.8% capacity after 300 cycles at 0.2 C, and 83.1% capacity after 200 cycles at 0.5 C at room temperature (Supplementary Fig. 19e and f). This excellent cycling performance is better than LFP/LLZTO/Li, LFP/LLZTO@Au/Li, and LFP/LLZTO@PEO/Li cells (Supplementary Fig. 20).

In summary, a flexible LiPAA EBS is formed between an LLZTO electrolyte and Li metal anode to suppress Li dendrite growth. Interfacial resistance is dramatically decreased from 1104.3 to 54.5 Ω cm^2^ at 25 °C due to a substitution reaction at the interface. The flexible EBS interface alleviates interfacial stress to maintain interfacial contact during cycling. The electronically insulating nature of the EBS is supported by electrostatic potential profiles and DOS results based on DFT simulations. EBS-protected LLZTO electrolytes prevent electronic degradation, avoiding the direct reduction of Li^+^ to Li metal dendrites within LLZTO. Li/LLZTO@EBS/Li cells exhibit a CCD as high as 1.2 mA cm^−2^ at 25 °C. Li/LLZTO@EBS/Li cells can continuously operate for over 1000 h at 0.2 mA cm^−2^ and 400 h at 1 mA cm^−2^. The performance of an EBS layer is superior to electron-conducting Au and traditional PEO polymer interfacial layers. This work represents the rational design of an interface for SSEs and Li metal anodes, and presents a promising strategy to achieve long-life and dendrite-free SSBs with high energy density and excellent safety.

## Methods

### Fabrication of LLZTO@PAA

Ta-doped garnet Li_6.4_La_3_Zr_1.4_Ta_0.6_O_12_ (LLZTO) powders were fabricated by the solid-state reactions, while LLZTO pellets were sintered by the hot-pressing technique^36^. The LLZTO ceramic pellets show the high relative density of 99.5 ± 0.5%, which was evaluated by the Archimedes’ principle (Supplementary Table 2 and Supplementary Note 3). PAA (average *M*_w_ ~450,000) polymer was dissolved in the DMSO solution by heating at 60 °C for 12 h. LLZTO@PAA was acquired by drip-casting the solution (0.25% PAA in DMSO) on the surface of LLZTO pellets and then vacuum drying at 80 °C.

### Material characterization

Crystal structures of samples were examined by XRD (Bruker D2 Phaser), using Cu Kα radiation with 2*θ* in the range of 10°–80° and a step size of 0.02°. Surface and cross-section morphologies of the LLZTO pellets were investigated by scanning electron microscopy (SEM, S3400). TOF-SIMS testing was conducted using TOF-SIMS IV (ION-TOF GmbH, Germany) with a 25 keV bismuth liquid metal ion source and a base pressure of ≈10^−8^ mbar in the analysis chamber. Negative secondary ions were induced by primary ion beam bombardment on the surface of LLZTO. The analysis area was 334 µm × 334 µm. Depth profiles were obtained by sputtering ion beams of Cs^+^ (3 keV) on a 100 µm × 100 µm square. Sputtering rate was measured on a Si wafer as 0.96 nm s^−1^ with a sputtered area of 100 µm × 100 µm. AFM (Dimension V equipped with a Nanoscope controller V and Nanoscope software 7.30, Veeco) was used to measure the elastic modulus of the LLZTO and LLZTO@PAA. The sensitivity and the spring constant of the AFM tip were measured under the contact model and thermal tune model, respectively. A force–strain mapping consisting of 10 × 10 points was measured in an area 10 μm × 10 μm. The elastic modulus mapping was fitted and plotted using the SPIP (Scanning Probe Image Processor) software. DEMS was used to detect the H_2_ release and confirm the reaction mechanism. The structure and composition of LiPAA studied by SEM, XPS, FTIR, and Raman.

To investigate the interface between the LLZTO ceramic pellets and Li metal, SEM sample preparation was performed as follows: the Li metal on the LLZTO pellets was melted at 250 °C for 30 min and then cooled to room temperature. The LLZTO pellets with Li metal were fractured using thin-tipped tweezers. Cross-sectional samples were chosen for SEM investigation.

To investigate the Li dendrite growing along the grain boundary of LLZTO ceramic pellets, SEM sample preparation was performed as follows: short-circuited cells were disassembled in an Ar-filled glovebox. After completely removing the Li metal on the LLZTO pellets by sanding, dark spots were observed on the white LLZTO surface, indicating the endpoints of Li dendrite penetration. LLZTO pellets were fractured at dark spots using thin-tipped tweezers. Cross-sectional samples with a black line along grain boundaries were chosen for SEM investigation.

### DFT calculations

Calculations were performed using the Vienna ab initio simulation package (VASP) code based on DFT^49,50^, employing the projector augmented wave (PAW) method as the potentials^51^, and the Perdew−Burke−Ernzerhof (PBE) generalized-gradient approximation (GGA) as the exchange-correlation functional^52^. In the model of PAA/Li, a single chain was designed to attach to Li(001), the Li atoms of the innermost layer are fixed to bulk Li, and a 35 Å vacuum slab was built on the Li surface. The DFT-D2 method was employed to incorporate van der Waals interactions between atoms^53^. The Li_2_CO_3_(001)/Li(001) and LLZO(110)/Li(001) models were built following refs. ^54–56^. For structural relaxation and energy/DOS calculations, an energy cutoff of 520 eV and a 1 × 1 × 1 K-point Monkhorst-Pack grid were used. The convergence criterion for energy and force for structural relaxation were set as 1.0 × 10^−5^ eV and 0.01 eV Å^−1^, respectively. All the structures were visualized by VESTA^57^.

### Electrochemical performance tests

Ionic conductivity of the LLZTO samples was measured by an impedance analyzer (Novocontrol Beta High Performance Impedance Analyzer) with an AC of 10 mV from 0.1 to 20 MHz in frequency. Thin gold layers were plated on both sides of the ceramic pellets by sputtering to be used as electrodes for conductivity testing. The LLZTO@PAA pellets were sandwiched between two pieces of Li metal to construct symmetric cells. Li metal electrodes were melted onto the two sides of the LLZTO@PAA pellets at 250 °C for 30 min in an Ar-filled glovebox before sealing in Swagelok-type cell molds. A pressure of ~10 N cm^−2^ was exerted on the ceramic plates using springs to maintain good contact. EIS measurements were performed in a frequency range from 1 MHz to 0.1 Hz with an amplitude of 10 mV by an Autolab instrument. Galvanostatic cycling tests were conducted using a NEWARE battery cycler (CT-4000) using different current densities at 25 °C. Li symmetric cells with other surface modifications were cycled under the same conditions.

The composite cathode was prepared as follows: 0.3 M Lithium bis(trifluoromethanesulfonyl)imide (LiTFSI, Sigma-Aldrich) was dissolved in ionic liquid (IL) (PY14TFSI, Sigma-Aldrich) to obtain a homogeneous IL-0.3 M solution. LiFePO_4_ (LFP), super P conductive additive (SP), polyvinylidene fluoride (PVDF), and IL-0.3 M with a weight ratio of LCO:SP:PVDF:IL-0.3 M = 8:1:1:6 were then ground thoroughly in a mortar. Finally, the slurry was coated on Al foil to form a composite cathode with an active material loading of ~2 mg cm^−2^.

## Supplementary information

Supplementary Information

## Data Availability

The data that support the findings of this study are available from the authors on reasonable request, see author contributions for specific data sets.
