# Supplementary material for: A flexible electron-blocking interfacial shield for dendrite-free solid lithium metal batteries
Source: Nat Commun. 2021 Jan 8;12:176. doi: 10.1038/s41467-020-20463-y (PMC7794502; doi:10.1038/s41467-020-20463-y)
Supplement: Supplementary file 1 — Supplementary Information [file 41467_2020_20463_MOESM1_ESM.pdf]

Supplementary Information

A flexible electron-blocking interfacial shield for dendrite-free solid lithium metal batteries

Hanyu Huo[1,2], Jian Gao[3], Ning Zhao[4], Dongxing Zhang[5], Nathaniel Graham Holmes[1], Xiaona Li[1], Yipeng Sun[1], Jiamin Fu[1], Ruying Li[1], Xiangxin Guo[4 *], Xueliang Sun[1 *]

[1] Department of Mechanical and Materials Engineering, University of Western Ontario, Ontario N6A 5B9, Canada
[2] State Key Laboratory of High Performance Ceramics and Superfine Microstructure, Shanghai Institute of Ceramics, Chinese Academy of Sciences, Shanghai 200050, China
[3] State Key Laboratory of Organic-Inorganic Composites, Beijing University of Chemical Technology, Beijing, 100029, China
[4] College of Physics, Qingdao University, Qingdao 266071, China
[5] Department of Chemistry, University of Western Ontario, Ontario N6A 5B9, Canada

[*] Corresponding authors.
Email address: xxguo@qdu.edu.cn, xsun9@uwo.ca

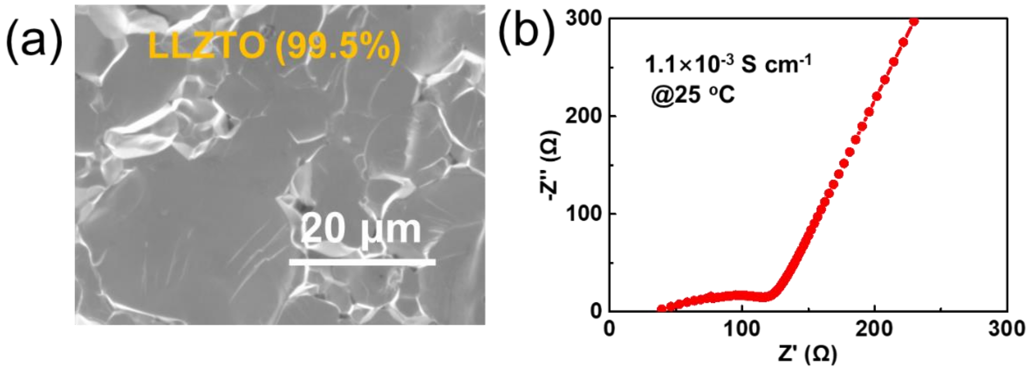

Supplementary Fig. 1 a) Cross-sectional SEM image, and b) EIS spectrum of the LLZTO pellet with a relative density over 99.5%.

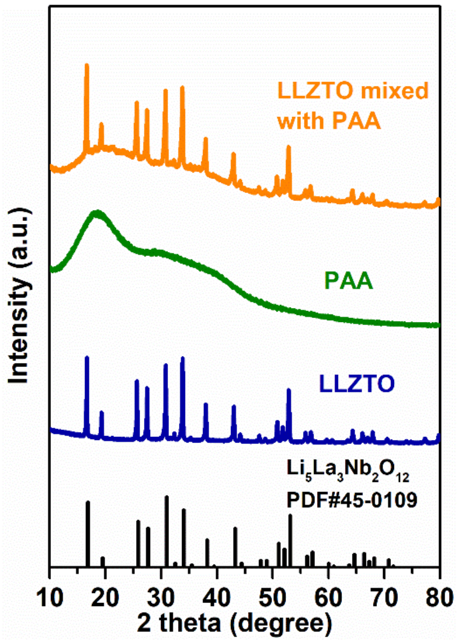

Supplementary Fig. 2 XRD patterns of a LLZTO pellet, PAA polymer, and LLZTO powder mixed with PAA.

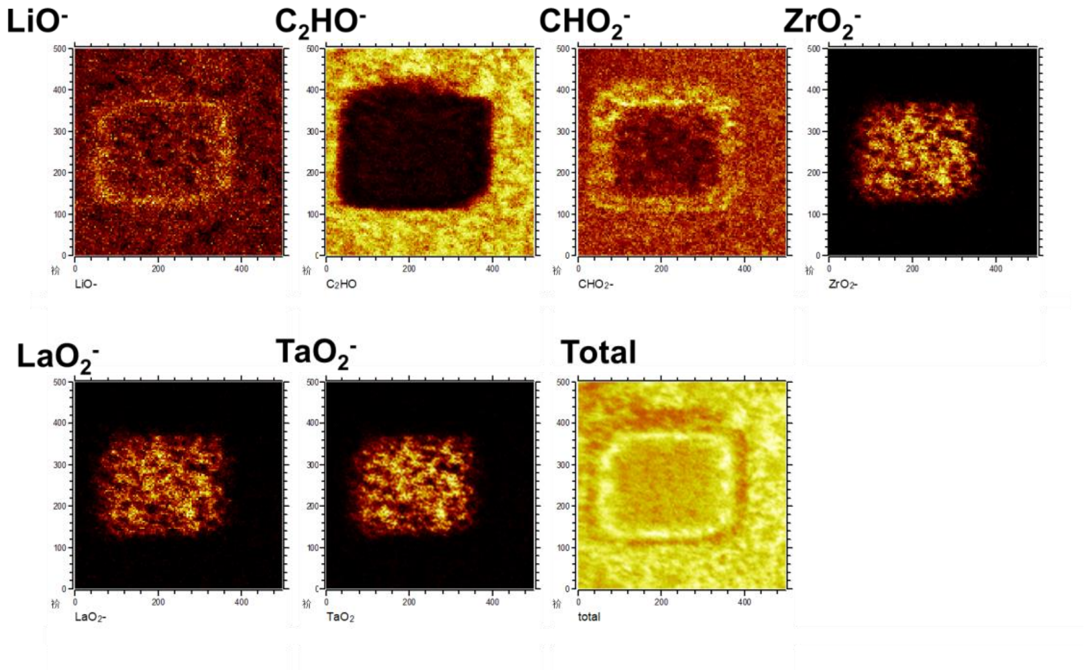

Supplementary Fig. 3 TOF-SIMS chemical mappings of LLZTO@PAA after sputtering.

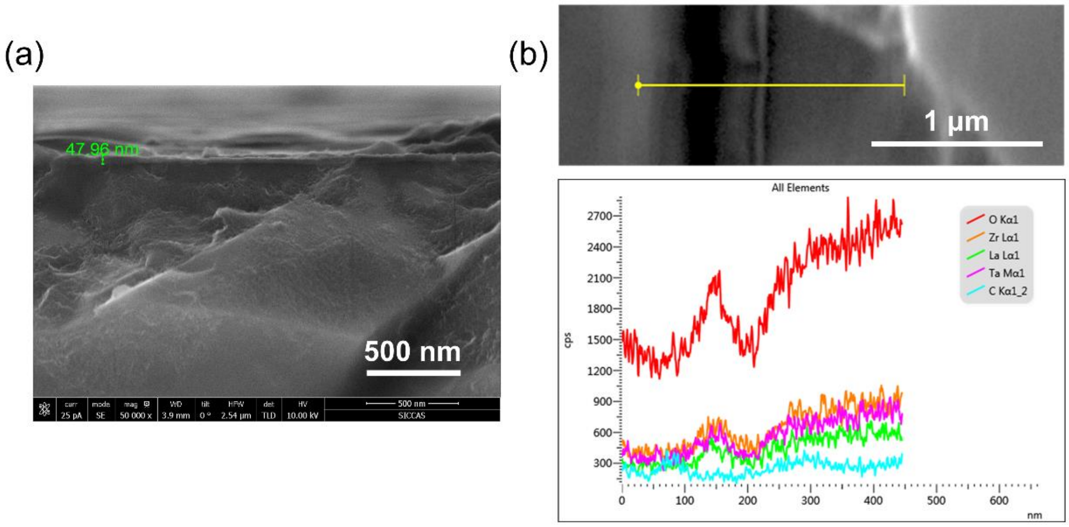

Supplementary Fig. 4 a) Cross-sectional SEM image and b) EDS of the PAA coating on the LLZTO.

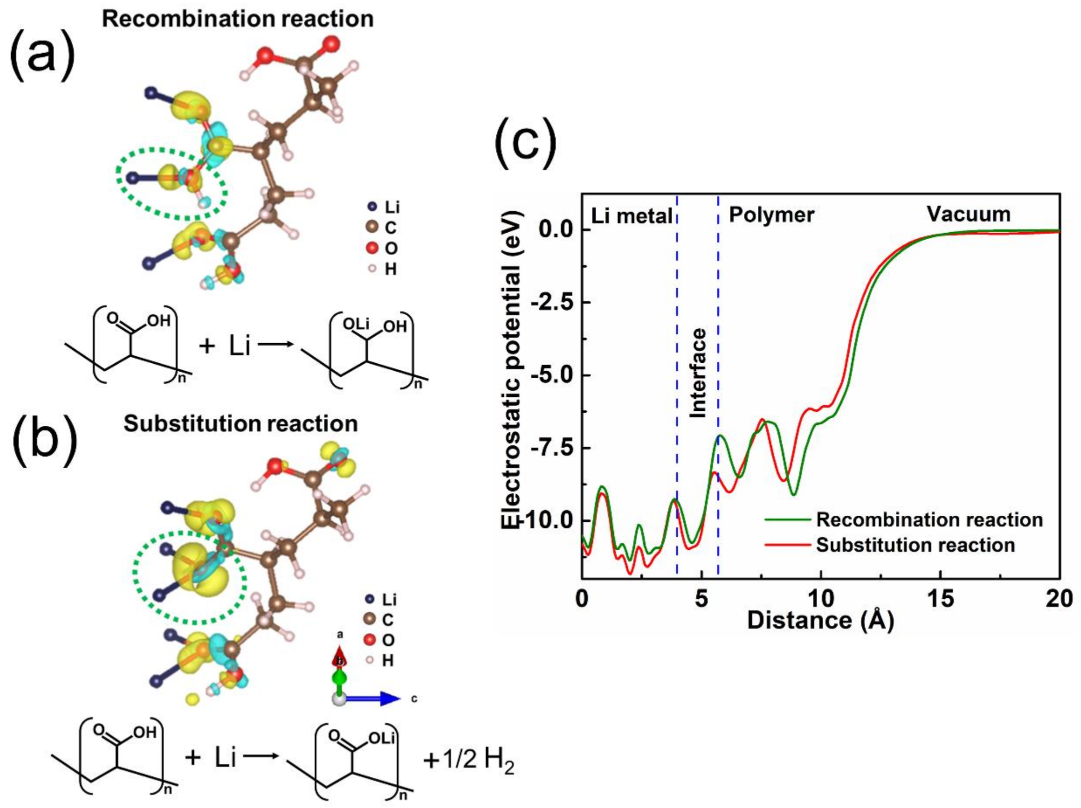

Supplementary Fig. 5 Partial PAA structures with single chain bonding with Li by a) a recombination reaction, and b) a substitution reaction. c) The electrostatic potential profiles for fully relaxed Li(001)/PAA with different reactions. The vacuum slab is not entirely presented

Supplementary Note 1. Gas detection during the reaction between the PAA and Li metal

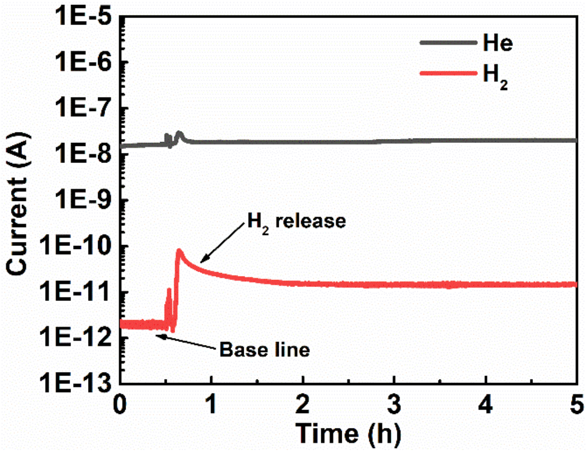

Supplementary Fig. 6 DEMS results of mass signals *m*/*z* 2 (H_2) and 4 (He).

Differential electrochemical mass spectrometry (DEMS) was used to detect the H_2 release and confirm the reaction mechanism. Two slices of Li foils were sandwiched by glass fiber separator, which was dropped by 80 μL PAA solution. As shown in Supplementary Fig. 6, the H_2 release confirms the substitution reaction between the -COOH group in the PAA and the Li metal.

Supplementary Note 2. Structure and composition of LiPAA

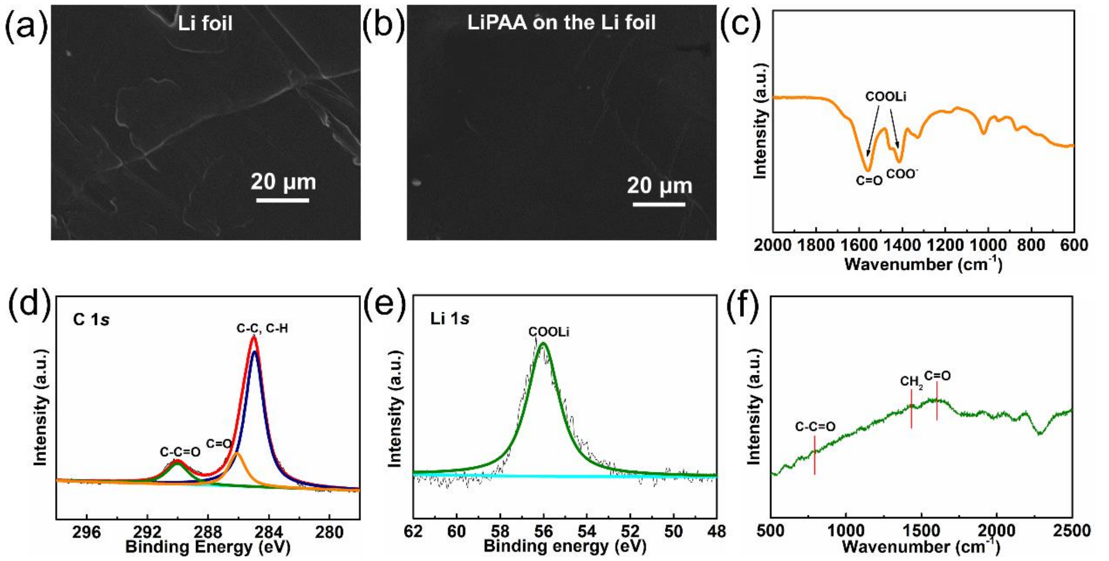

Supplementary Fig. 7 Top-view SEM images of a) the Li foil and b) the LiPAA on the Li foil. c) FT-IR of the LiPAA on the Li foil. d) C 1*s* and e) Li 1*s* XPS spectra of the LiPAA on the Li foil. f) Raman spectrum of the LiPAA on the Li foil.

The structure and composition of LiPAA was investigated by various characterizations to support the results of theoretical calculations. The Li metal was immersed into the PAA solution. The formed LiPAA on the surface of the Li metal is easier to study than that sandwiched between the LLZTO and the Li metal. The surface of Li metal becomes smoother due to the LiPAA coating layer (Supplementary Fig. 7a and 7b). The typical COOLi group in the LiPAA is confirmed by the FTIR, XPS, and Raman tests (Supplementary Fig. 7c-7f).

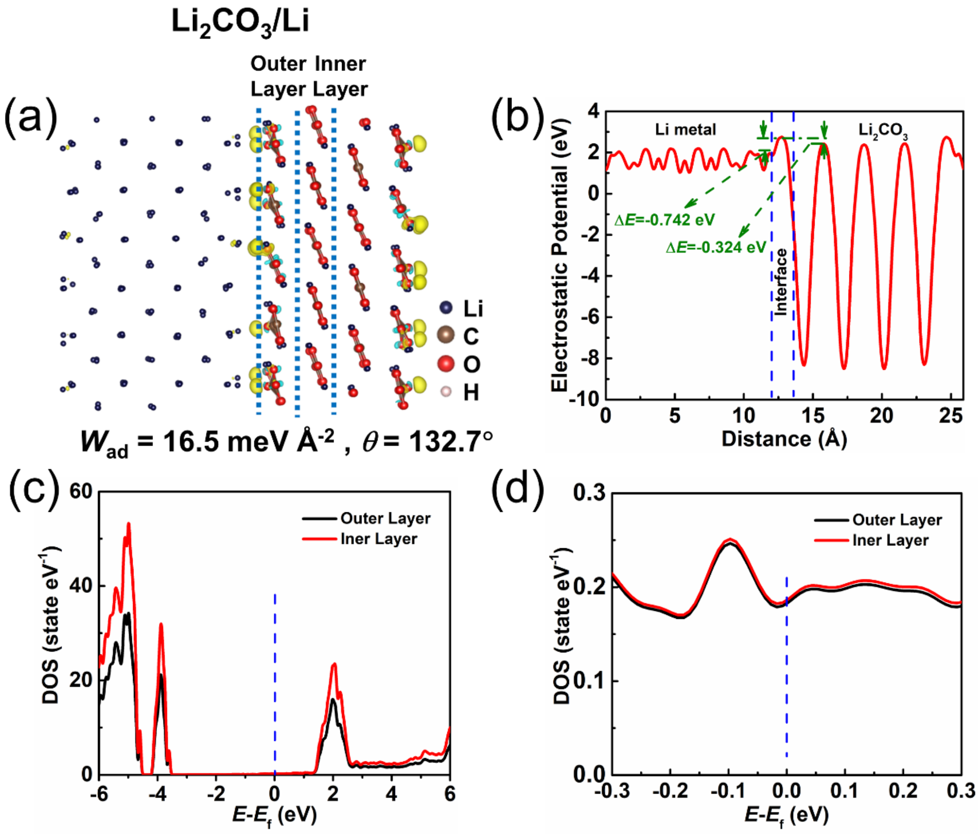

Supplementary Fig. 8 a) The structure and charge transfer, b) the electrostatic potential profiles, and c,d) the DOS for fully relaxed Li(001)/Li_2CO_3(001).

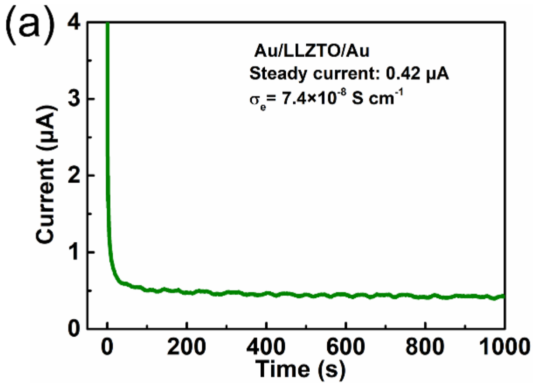

Supplementary Fig. 9 Current-time curves of a) the Au/LLZTO/Au cell and b) the Au/LLZTO@EBS/Au cell under DC polarization at 0.1 V.

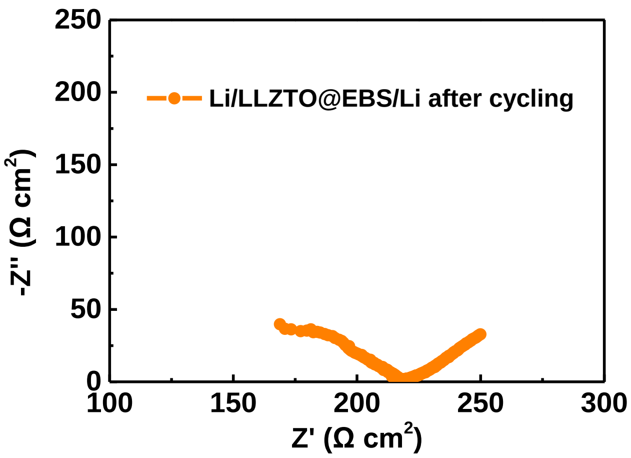

Supplementary Fig. 10 EIS spectrum of a Li/LLZTO@EBS/Li cell after cycling at 0.2 mA cm^{-2} (0.1 mAh cm^{-2}) for 1000 h.

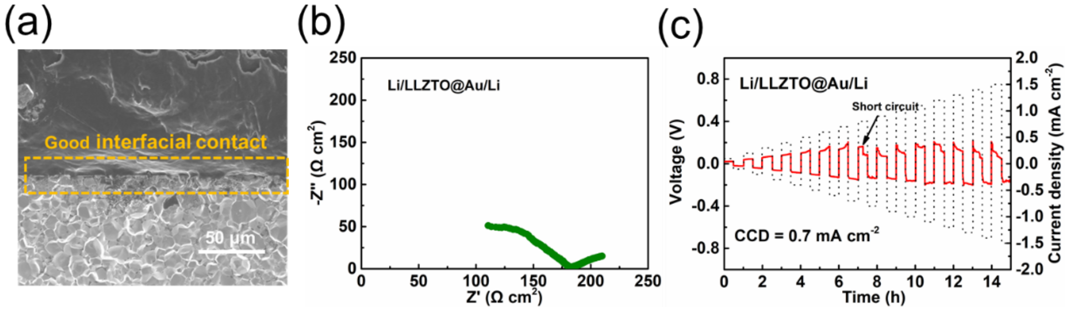

Supplementary Fig. 11 SEM image of the LLZTO@Au/Li interface. b) EIS spectrum, and c) CCD of a Li/LLZTO@Au/Li cell at 25 ºC.

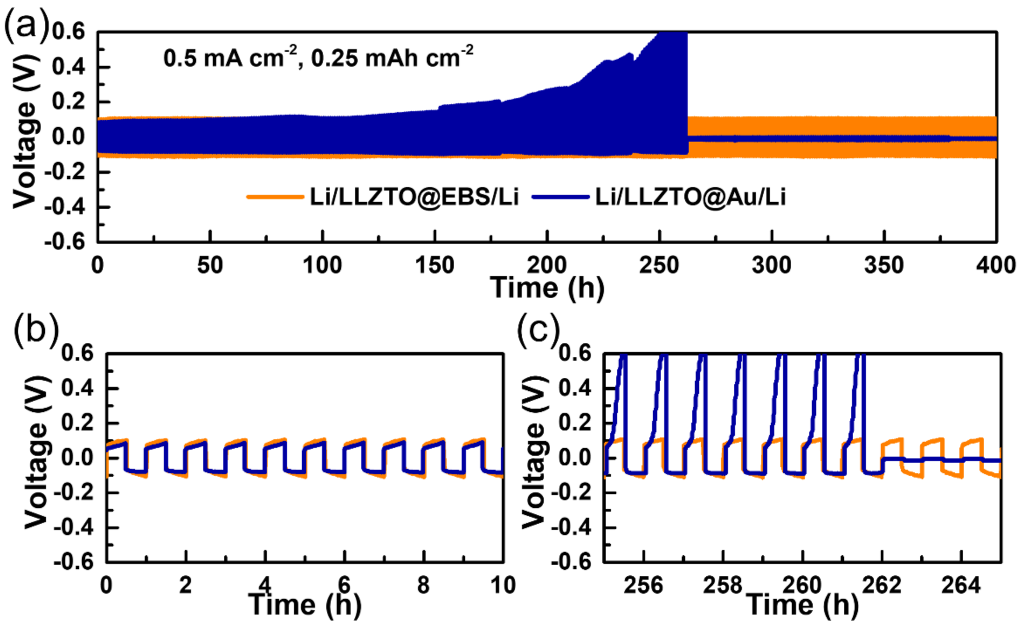

Supplementary Fig. 12 a) Galvanostatic cycling performance of the Li/LLZTO@Au/Li and Li/LLZTO@EBS/Li cells at 0.5 mA cm^{-2} (0.25 mAh cm^{-2}) at 25 ºC. Magnified images for b) 0-10 h, and c) 255-265 h.

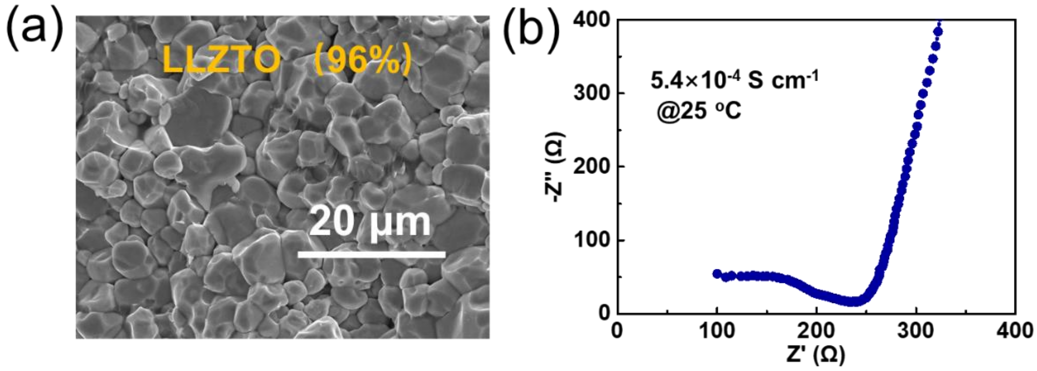

Supplementary Fig. 13 a) Cross-sectional SEM image, and b) EIS spectrum of a LLZTO pellet with a relative density of 96%.

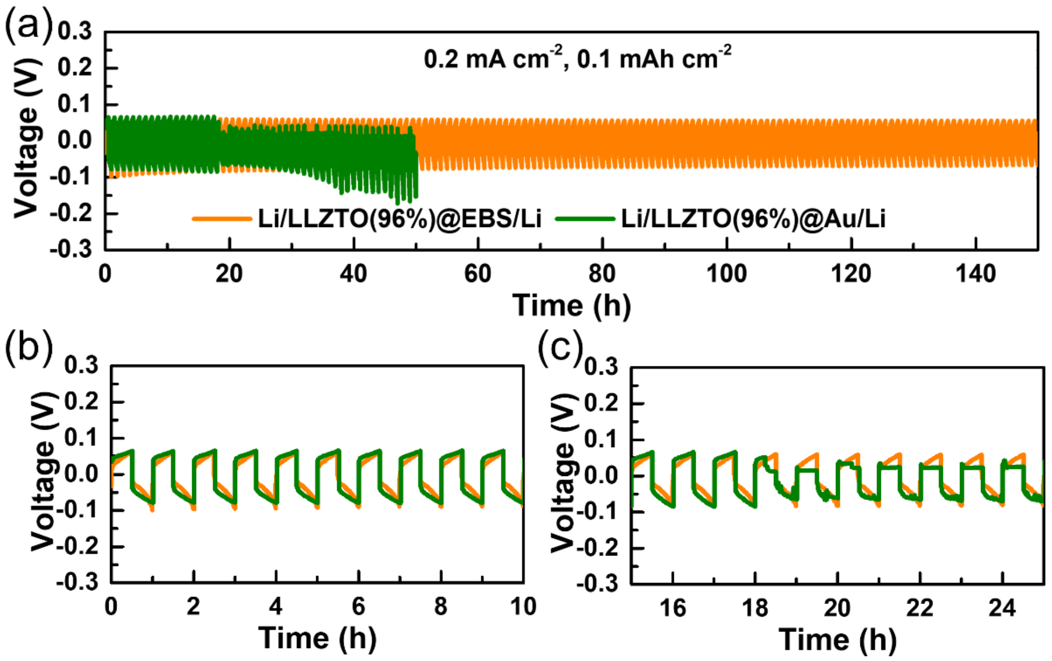

Supplementary Fig. 14 a) Galvanostatic cycling performance of the Li/LLZTO(96%)@Au/Li and Li/LLZTO(96%)@EBS/Li cells at 0.2 mA cm^{-2} (0.1 mAh cm^{-2}) at 25 ºC. Magnified images for b) 0-10 h, and c) 15-25 h.

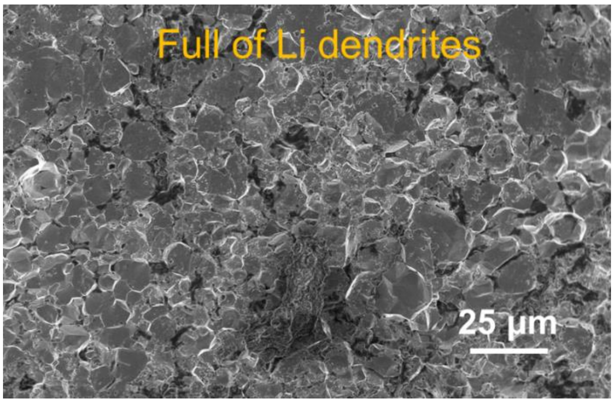

Supplementary Fig. 15 Cross-sectional SEM image of LLZTO(96%)@Au after short circuiting.

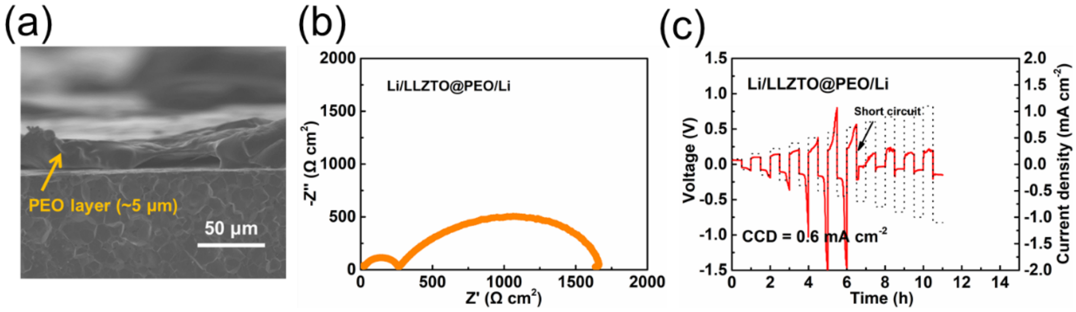

Supplementary Fig. 16 a) SEM image of the PEO electrolyte layer on the surface of a LLZTO pellet. b) EIS spectrum, and c) CCD of a Li/LLZTO@PEO/Li cell at 60 ºC

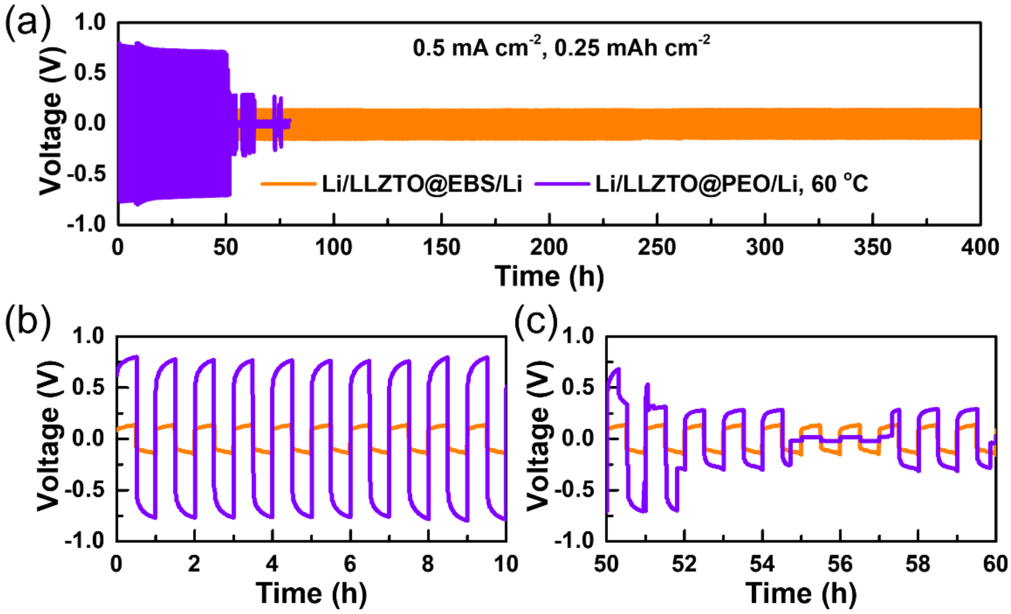

Supplementary Fig. 17 a) Galvanostatic cycling performance of the Li/LLZTO@PEO/Li and Li/LLZTO@PEO/Li cells under 0.5 mA cm^{-2} (0.25 mAh cm^{-2}). Magnified images for b) 0-10 h, and c) 50-60 h. The working temperatures for the Li/LLZTO@PEO/Li and Li/LLZTO@EBS/Li cells are 60 and 25 ºC, respectively.

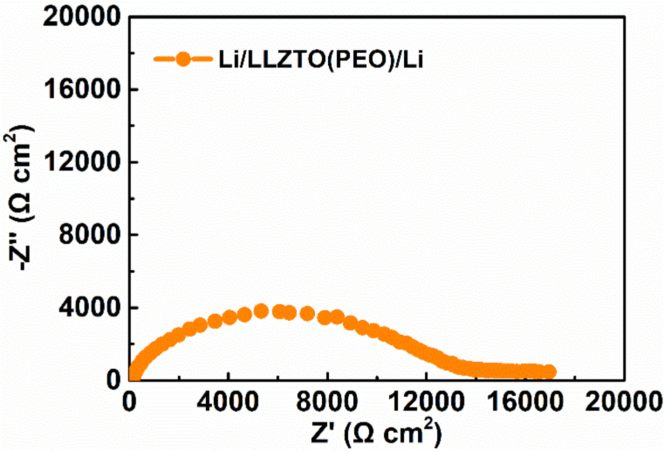

Supplementary Fig. 18 EIS spectrum of the Li/LLZTO(PEO)/Li cell.

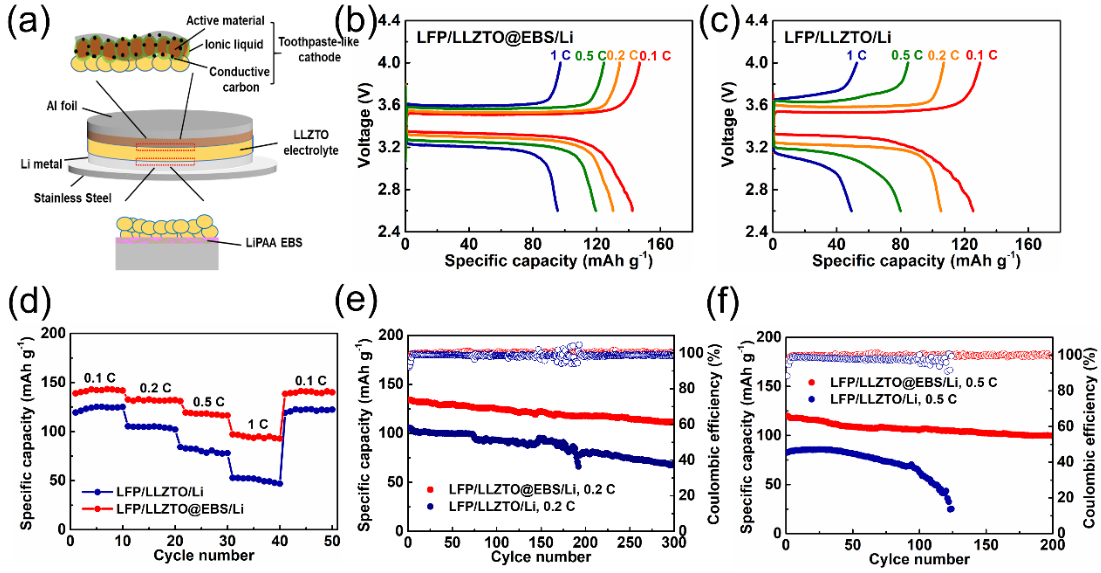

Supplementary Fig. 19 a) Schematic representation of SSBs with a Li metal anode, an LLZTO@EBS electrolyte, and an LFP cathode. Charge/discharge curves of the b) LFP/LLZTO@EBS/Li, and c) LFP/LLZTO/Li cells at various current rates. d) Rate performance of the LFP/LLZTO@EBS/Li and LFP/LLZTO/Li cells. e) Cycle performance of the LFP/LLZTO@EBS/Li and LFP/LLZTO/Li cells under e) 0.2 C, and f) 0.5 C at room temperature.

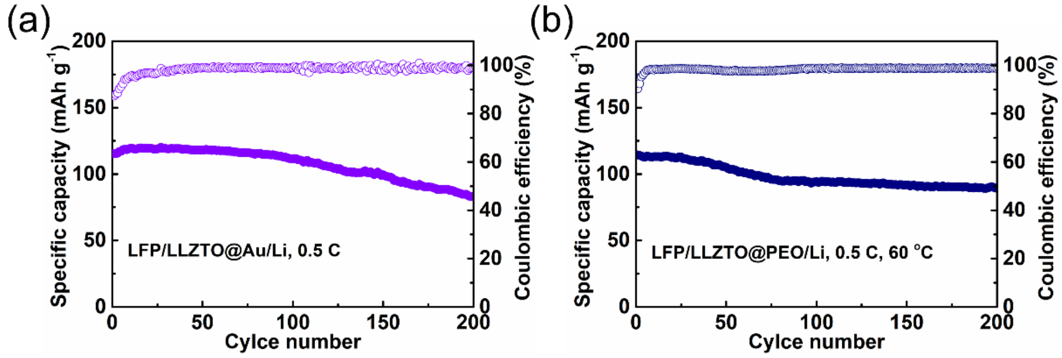

Supplementary Fig. 20 a) Cycling performance of a LFP/LLZTO@Au/Li cell at a) 0.5 C at room temperature. b) LFP/LLZTO@PEO/Li cells at 0.2 C at 60 ºC.

Supplementary Table 1 Comparison of critical current density (CCD) and cycling stability between our work and recent publications.

Interfacial modification	Interfacial resistance (Ω cm^2)	CCD (mA cm^{-2})	Stability (RT) (mA cm^{-2} /lifetime in h)	Ref
Bare LLZTO	1104.3	0.2	0.2/2	
Sputtering Au	101.6/167.8	0.5	0.08/150	1
ALD Al_2O_3	34	N/A	0.2/90	2
PECVD Si	127	0.2	0.1/225	3
ALD Al	75	0.2	0.2/42	4
Drawing Soft graphite	105	N/A	0.3/1000	5
Co-sputtering Cu_6Sn_5	236	N/A	0.25/300	6
Polishing MoS_2	14@100 °C	2.2@100 °C	0.8/240@100 °C	7
PECVD Li_3N	175	N/A	0.1/210	8
Sputtering Cu_3N	83.4	1.2	0.5/400	9
Dip-casting PAA	54.5	1.2	1/400	This work

Supplementary Note 3. The relative density of LLZTO ceramic pellets evaluated by Archimedes' principle

Supplementary Table 2 Two groups of relative density data

	m_{LLZTO}	$\rho_{ethanol}$	$m_{submerged}$	ρ_{real}	$\rho_{theoretical}$	$\rho_{relative}$
Group1	0.598 g	0.789 g cm^{-3}	0.512 g	5.486 g cm^{-3}	5.5 g cm^{-3}	99.7%
Group2	0.578 g	0.789 g cm^{-3}	0.495 g	5.494 g cm^{-3}	5.5 g cm^{-3}	99.8%

The relative density of LLZTO ceramic pellets was evaluated by the Archimedes' principle, which is shown as the following formula,

$$\rho_{relative} = \rho_{real}/\rho_{theoretical} \times 100\% \quad (1)$$

$$\rho_{real} = m_{LLZTO} \bullet \rho_{ethanol}/(m_{LLZTO} - m_{submerged}) \quad (2)$$

Where the $\rho_{relative}$ is the relative density of the LLZTO, the ρ_{real} and $\rho_{theoretical}$ are the tested density and the theoretical density of the LLZTO, m_{LLZTO} is the mass of the LLZTO, $m_{submerged}$ is the apparent mass of the LLZTO when submerged in the ethanol, and $\rho_{ethanol}$ is the density of the ethanol. Two groups of relative density data are shown in Supplementary Table 2. Note that the thicknesses of LLZTO pellets for relative density tests are approximately 0.3 mm in order to decrease the effects of closed pores. It was found that various thicknesses of LLZTO pellets show no influences on the testing results, indicating the excellent uniformity in the ceramic pellets fabricated by hot-pressing method. The tested $\rho_{relative}$ has ±0.5% error.

Supplementary References

1. Tsai, C.-L. *et al.* $Li_7La_3Zr_2O_{12}$ Interface Modification for Li Dendrite Prevention. *ACS Applied Materials & Interfaces* **8**, 10617-10626 (2016).
2. Han, X. *et al.* Negating interfacial impedance in garnet-based solid-state Li metal batteries. *Nat Mater* **16**, 572-579 (2017).
3. Luo, W. *et al.* Transition from Superlithiophobicity to Superlithiophilicity of Garnet Solid-State Electrolyte. *J. Am. Chem. Soc.* **138**, 12258-12262 (2016).
4. Fu, K. K. *et al.* Toward garnet electrolyte–based Li metal batteries: An ultrathin, highly effective, artificial solid-state electrolyte/metallic Li interface. *Science Advances* **3**, e1601659 (2017).
5. Shao, Y. *et al.* Drawing a Soft Interface: An Effective Interfacial Modification Strategy for Garnet-Type Solid-State Li Batteries. *ACS Energy Lett.* **3**, 1212-1218 (2018).
6. Feng, W. L. *et al.* Building an Interfacial Framework: Li/Garnet Interface Stabilization through a Cu_6Sn_5 Layer. *Acs Energy Letters* **4**, 1725-1731 (2019).
7. Fu, J. M. *et al.* In situ formation of a bifunctional interlayer enabled by a conversion reaction to initiatively prevent lithium dendrites in a garnet solid electrolyte. *Energy Environ. Science* **12**, 1404-1412 (2019).
8. Xu, H. *et al.* Li_3N-Modified Garnet Electrolyte for All-Solid-State Lithium Metal Batteries Operated at 40 degrees C. *Nano Lett.* **18**, 7414-7418 (2018).
9. Huo, H. Y. *et al.* Design of a mixed conductive garnet/Li interface for dendrite-free solid lithium metal batteries. *Energy Environ. Science* **13**, 127-134 (2020).
